# A novel affordable reagent for room temperature storage and transport of fecal samples for metagenomic analyses

**DOI:** 10.1186/s40168-018-0429-0

**Published:** 2018-02-27

**Authors:** Mo Han, Lilan Hao, Yuxiang Lin, Fang Li, Jian Wang, Huanming Yang, Liang Xiao, Karsten Kristiansen, Huijue Jia, Junhua Li

**Affiliations:** 1 BGI-Shenzhen, Shenzhen, 518083 China; 2 China National Genebank, BGI-Shenzhen, Shenzhen, 518120 China; 3 Department of Biology, Laboratory of Genomics and Molecular Biomedicine, University of Copenhagen, Universitetsparken 13, 2100 Copenhagen, Denmark; 4 James D. Watson Institute of Genome Sciences, Hangzhou, 310058 China; 5 School of Bioscience and Biotechnology, South China University of Technology, Guangzhou, 510006 China; 6 Shenzhen Key Laboratory of Human commensal microorganisms and Health Research, BGI-Shenzhen, Shenzhen, 518083 China; 7 Shenzhen Engineering Laboratory of Detection and Intervention of Human Intestinal Microbiome, Shenzhen, 518083 China

**Keywords:** Fecal sample, Room temperature storage, Room temperature transport, Metagenomic sequencing, *N*-octylpyridinium bromide

## Abstract

**Background:**

The number of large-scale studies on the gut microbiota in human cohorts is rapidly increasing. However, the few and expensive options for storage of fecal samples at room temperature have been an obstacle for large-scale metagenomic studies and the development of clinical/commercial personal metagenomic sequencing.

**Results:**

In this study, we systematically tested a novel *N*-octylpyridinium bromide-based fecal sample preservation method and compared it with other currently used storage methods. We found that the *N*-octylpyridinium bromide-based method enabled preservation of the bacterial composition in fecal samples transported and stored at room temperature for up to at least 14 days.

**Conclusions:**

We describe a novel chemical stabilizer that allows cost-effective transportation and storage at room temperature for several days with preservation of bacterial composition. This method will facilitate sample collection even in remote area and also enable transport via normal commercial transportation routes.

**Electronic supplementary material:**

The online version of this article (10.1186/s40168-018-0429-0) contains supplementary material, which is available to authorized users.

## Background

Recent advances in sequencing technics and bioinformatics, especially in the field of metagenomic sequencing, have increased our knowledge of the complex microbial communities in the gut of humans and animals. It is now well established that these microbes play important roles in relation to inflammation [1], metabolic disease [2, 3], mental disorders [4, 5], and several other diseases [6–8]. Studies of the gut microbiota may help us to prevent, diagnose, and eventually cure or curb such diseases [9, 10].

Fecal samples are widely used in metagenomic studies and are generally required to be immediately stored at − 20 °C or below. However, this is difficult to achieve in many situations such as sampling in remote areas and may dramatically increase the costs of such studies. Few studies have in detail investigated the stability of fecal samples, and in many cases, samples have been stored at room temperature for several days prior to storage at − 20 °C or below [11–14]. Chelating agent-based solutions are used to stabilize nucleic acid contained in feces at ambient temperature, such as the widely used commercial fecal sampling kit, OMNIgene•GUT OM-200 (DNA Genotek Inc.). Although the performance has been tested [15, 16], the relatively high cost (about $20 per sample) often comes between the kits and large-scale applications. To overcome the problems associated with sampling without possibilities for immediate storage at − 20 °C or below and transportation, and secure the generation of standardized and reproducible metagenomic sequencing data quality, we developed and tested a fecal sample preservation protocol using storage in a novel *N*-octylpyridinium bromide (NOPB)-based reagent.

To evaluate the NOPB-based method, we collected ten fecal samples from eight healthy adult subjects and divided these samples into 110 aliquots. The aliquots were stored or transported at room temperature using different schemes before extraction. After storage and/or transportation, all aliquots were sequenced, and each was compared to the corresponding freshly extracted sample. Aliquots which have been stored without any additional reagents (termed “non-stabilized”) were used for further comparison. Extracted DNA from all samples was sequenced on Illumina HiSeq 4000, and a subset was also sequenced on the BGISEQ-500 instrument.

## Methods

### Reagents and kits

Two reagents/kits were used in this study: the Genotek OMNIgene·GUT OM-200 (DNA Genotek Inc., Canada), which provides a chelating agent-based solution for stabilizing nucleic acid contained in feces for collection, storage, and transportation at ambient temperature, and the novel NOBP-based reagent. The composition of the NOBP-based reagent is given in Table 1.

**Table 1:** The composition of NOBP-based stabilizer reagent

Components	Concentration
Lithium chloride (LiCl)	3.5 M
Tris (hydroxymethyl) aminomethane (Tris)	200 mM
Ethanol	30% (*v*/*v*)
Tris (2-carboxyethyl) phosphine hydrochloride (TCEP·HCl)	20 mM
*N*-Octylpyridinium bromide	4% (*v*/*v*)

The pH of the reagent was adjusted to 8.0 with NaOH

### Sample collection and treatment

**Fig. 1:**
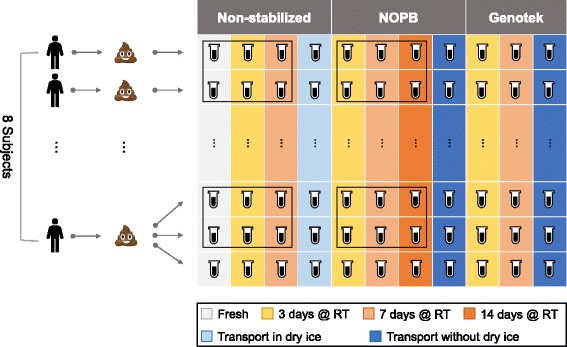
Layout of sampling and treatment. All aliquots except the “14 days @ RT” group were sequenced on the HiSeq 4000 platform, while the aliquots in the black frames were also sequenced on the BGISEQ-500 platform

Eight subjects provided fecal samples in a stool container after having signed an informed consent agreement. The samples were placed in ice boxes and delivered to the laboratory within 5 min. Each of seven samples was divided under anaerobic conditions into 11 aliquots (77 aliquots in total), each weighing about 200 mg, while the eighth sample was first divided into three parallel samples for replication, and then into 33 aliquots. For each 11 aliquots from the same sample, one aliquot was taken as the fresh control, representing the baseline composition of the sample, and DNA was extracted immediately; four of the aliquots were placed into 2.0-ml centrifuge tubes with 500 μl of the NOPB-based stabilizer reagent; three were treated by the commercial self-collection kit OMNIgene·GUT OM-200 following the instructions of the manufacturer; and the last three aliquots were stored in 2.0-ml centrifuge tubes without any additions. To test how shipment affected the stability of the samples, 2.0-ml centrifuge tubes containing aliquots of NOPB-based reagent-treated and OM-200-treated samples were placed in a styrofoam chest without dry ice, while non-stabilized fresh samples which had been stored at − 80 °C were placed in a styrofoam chest filled with dry ice. The two chests were then transported to Guangzhou from Shenzhen and back to Shenzhen using a commercial provider (SF Express Inc., China). The transportation took 3 days. Details on how the samples were aliquoted and handled are illustrated in Fig. 1.

### DNA extraction

DNA was extracted as previously described with minor modifications [2]. Aliquots were centrifuged at 12,000 rpm for 5 min, resuspended in PBS, and then centrifuged again at 12,000 rpm for 5 min. The pellet was used for extraction, and the supernatant was discarded. The pellet was resuspended in 250 μl of 4 M guanidine thiocyanate-0.1 M Tris (pH 7.5) and 40 μl of 10% *N*-lauroyl sarcosine. After that, 500 μl of 5% *N*-lauroyl sarcosine was added and the sample was incubated in 70 °C for 1 h. After incubation, 500 μl of glass beads (0.1 mm) and 500 μl of TENP were added into the tube. After vortexing at full speed for 15 min, the sample was centrifuged at 13000 rpm for 15 min. Nine hundred microliters of supernatant was transferred to a new tube, and the DNA was precipitated by 900 μl of isopropanol. For purifying, DNA was resuspended by PBS and precipitated again with ethanol and washed. After all, 2 μl of RNase A was added to the 80 μl of DNA-TE solution to digest the RNA before storing the sample into a freezer.

### Library construction and sequencing

Sequencing was performed using either the Illumina HiSeq platform or the BGISEQ-500 platform, which was recently validated and benchmarked against the Illumina HiSeq platform [17]. Sequencing libraries for Illumina HiSeq sequencing were prepared following the manufacturer’s instructions as described previously [2]. We constructed Illumina libraries for 100 aliquots, followed by sequencing to obtain about 30 million single-end (SE) reads per aliquot on the HiSeq 4000 sequencer (named as dataset A). Twenty-four aliquots were used to construct sequencing libraries for the BGISEQ-500 sequencer as described [17] to obtain about 30 million SE reads per aliquot (named as dataset B).

The read length was 50 bp for both datasets A and B. Raw reads were quality controlled by filtering low-quality reads, adapter contamination, and/or human DNA contamination before analysis. Briefly, FASTX Toolkit was used for quality control, while SOAPaligner2 and the hg19 genome were used for identifying human sequences as described before [18].

### Abundance calculation and taxonomic annotation

Relative abundances of genes were determined by aligning the high-quality reads to the gut microbial reference catalog of 9,879,896 genes as described [18] with a cut-off of relative abundance at 1.0 × 10^− 8^. The phylogenetic annotation pipeline ensured unique assignment to phyla, genera, and species as previously described [19].

### Calculation of the correlation coefficient and dissimilarity

Spearman index calculated in R (3.4.1, *vegan* package) was used to determine correlation coefficients between the relative abundance profiles of aliquots. R (3.4.1, *dist* function) was also used to calculate Euclidean distances and Bray-Curtis dissimilarity. To test for differences in correlation coefficients and dissimilarities among groups, paired *t* test was used. The *q*-*q* plots and the box plot of the trendlines were also plotted in R (3.4.1, *ggplot2*).

### Biodiversity analysis

To estimate gut microbial diversity, we calculated the α-diversity using the Shannon-Wiener’s index at the gene, genus, and species level as previously described [18] in R (3.4.1, *vegan* package). To test for differences in biodiversity among groups, paired *t* test was used.

### Variance analyzing

To discover genes or genera exhibiting differences in abundance between samples stored according to the different methods, paired *t* test between each two of the groups was used. We applied the FDR method proposed in a previous study [20] to adjust the *p* value.

Principal co-ordinate analysis (PCoA) was performed by R (3.4.1, *ade4* package) based on both Euclidean distance and Bray-Curtis dissimilarity; PERMANOVA was applied to test for significant differences between groups.

## Results

### Similarity between stored and fresh fecal samples

Firstly, Good’s coverage of each aliquot was calculated on genes level. The result (mean = 0.9921, s. d. = 0.0020) suggested the depth of sequencing is enough and uniform. Afterwards, we compared how storage with no stabilizer and with the NOBP stabilizer for different length of time affected the composition of the bacterial composition at the gene, genus, and species level compared with freshly extracted samples. This analysis showed that alpha diversity as measured by the Shannon-Wiener index at the gene, genus, and species levels of samples stored in the NOPB-based stabilizer did not deviate from those of freshly extracted samples even after storage for up to 14 days, whereas deviations as expected were observed for samples with no added stabilizer. Distance and dissimilarity between samples stored in the NOPB-based and freshly extracted samples were significantly lower than those observed in analyzing the non-stabilized samples, although the correlation coefficient measured by the Spearman index did not show significant difference between the two protocols (Additional file 1: Figure S1).

**Fig. 2:**
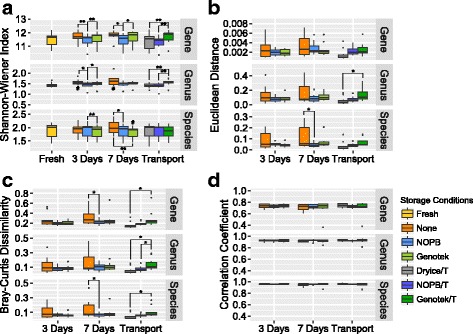
Correlation, dissimilarity, and change in α-diversity between stored or transported and fresh fecal samples. Sequencing data (dataset A) used to plot this figure was generated by using the Illumina HiSeq 4000 platform. “/T” represents transportation groups. **a** The α-diversity of the freshly extracted samples and corresponding stored samples. **b**–**d** The Euclidean distance, Bray-Curtis dissimilarity, and Spearman correlation coefficient between the stored samples and corresponding freshly extracted ones. One asterisk indicates significant difference (*p* < 0.05, paired *t* test), and two asterisks indicate highly significant difference (*p* < 0.01, paired *t* test). Number sign in **d** indicates significant difference in α-diversity in comparison to the corresponding fresh aliquots (*p* < 0.05, paired *t* test) (*n* = 10)

Compared with the corresponding fresh samples, aliquots stored in the NOPB-based stabilizer reagent and the samples kept at − 80 °C and transported in dry ice showed significantly lower dissimilarity in relation to relative gene abundances and microbiome composition than those in the non-stabilized samples after 7-day storage (Fig. 2b, c). Aliquots stabilized by the NOPB-based reagent or transported on dry ice exhibited the highest correlation with the fresh samples as well as lowest dissimilarity in the transportation test. Of note, our NOPB-based stabilizer actually showed better performance in comparison to the OM-200 kit when the aliquots were transported. The Bray-Curtis dissimilarity between samples collected and transported using the OM-200 kits and corresponding fresh samples was significantly higher than that of the dry ice group, indicating that the relative abundances of abundant genes/taxa in the samples transported using the OM-200 kits were changed, whereas we observed no significant difference between samples shipped in the NOPB-based reagent or on dry ice (Fig. 2c). Furthermore, we observed the dissimilarity, and α-diversity of the non-stabilized aliquots differ significantly from samples stabilized by using the NOPB reagent in most of the tests, while the distance and dissimilarity between non-stabilized stored aliquots and the corresponding freshly extracted samples exhibited greater variation (Fig. 2).

We compared the effect of storage time for samples stored in the absence or presence of a stabilizer. As expected, we found that samples stored in the NOPB reagent and the OM-200 reagent exhibited lower variation over time than the non-stabilizer samples (Additional file 1: Figure S2).

Further PCoA analyses at the gene, genus, and species level comparing all storage conditions revealed minor differences between the different storage conditions. Generally, samples stored or transported use the NOPB-based method and those kept and transported in dry ice clustered slightly closer to the corresponding freshly extracted samples than the non-stabilized samples as well as the samples stored or transported using OM-200 kit (Additional file 1: Figure S3). However, we found no statistically significant differences between any of the methods.

### Biases of different methods for storage

**Fig. 3:**
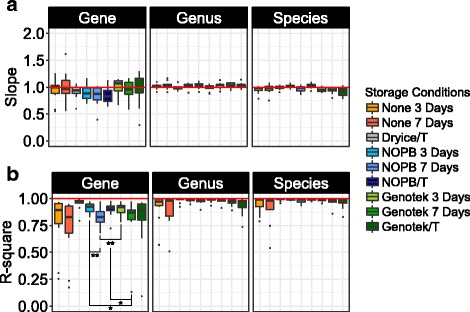
Bias and variation of different storage conditions related to relative abundance. **a** The slopes of the linear trendlines in the *q*-*q* plot. **b** The *R*-square values. One asterisk indicates significant difference (*p* < 0.05, Wilcoxon test), and two asterisks indicate highly significant difference (*p* < 0.01, Wilcoxon test)

We plotted the *q*-*q* plot of each storage condition to illustrate the dissimilarities in composition at the level of genes and/or taxa between the stored aliquots and the corresponding fresh aliquots (Additional file 1: Figures S4 and S5). The slope of the trendline and the *R*-square value of each storage condition were used to estimate the bias and variation in comparison to the original relative abundances at the gene, genus, and species level. The results indicate that samples stored in the NOPB-based reagent exhibited a slightly better performance in relation to recovery of low abundance species and exhibited a lower variation than observed for samples stored using the OM-200 kits, especially when samples had been transported (Fig. 3).

There were no significant differences in relative gene abundances comparing samples stored and transported in the NOPB-based stabilizer reagent with the freshly extracted samples. However, aliquots collected in the OM-200 reagent and transported exhibited some differences at the genus and species level when compare to samples transported using dry ice, while such differences were not observed for the aliquots transported in the NOBP stabilizer reagent (Table 2).

**Table 2:** Taxa exhibiting significant difference in abundance in the transportation test

Genotek/T vs. dry ice/T	Genus	Fold change	Adjusted *p*
	*Pseudoflavonifractor*	1.412	0.026
	Species	Fold change	Adjusted *p*
	*Clostridium saccharolyticum*	1.455	0.034
	*Pseudoflavonifractor capillosus*	1.521	0.034
	*Lachnospiraceae oral taxon 107*	1.738	0.034
	Unclassified *Ruminococcaceae bacterium D16*	1.865	0.049

**Fig. 4:**
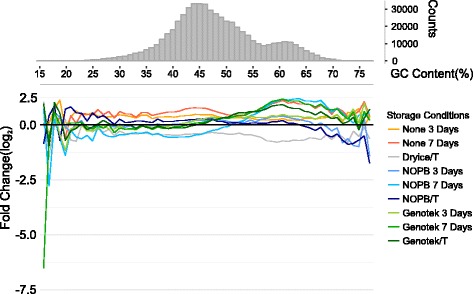
Biases of different storage conditions related to GC content. Lines show the mean fold change of genes with different GC content (window size = 1%). Bars indicate counts of genes in relation to GC content. “/T” represents transportation groups

We evaluated possible biases due to different GC content of genes. The analyses revealed that both the NOPB and the OM-200 kit exhibited slight biases towards high GC content, but the average fold change did not deviate from samples stored in dry ice (Fig. 4).

## Discussion

A reliable and cost-efficient method which maintains relative gene abundances and microbiota composition of fecal samples during storage and/or transportation at room temperature is highly desirable for large-scale metagenomic studies. The NOPB-based method represents such a reliable choice. Our results demonstrate that fecal samples which have been stored for up to 2 weeks do not deviate from freshly extracted samples in connection with metagenomic sequencing. The NOPB-based protocol showed slightly better performance compared with another currently and commonly used protocol for sampling and storage of fecal samples at room temperature, especially when samples were transported using normal commercial services. Therefore, the NOPB-based stabilized enables more easy sampling in connection with large cohort studies and from individuals living in remote difficult to access areas, improving possibilities and securing high quality of the collected samples. Furthermore, since the NOPB-based fecal sample preservation method did not cause any PCR inhibition during the library construction procedure nor other experiments we did, a combination of the method and amplicon sequencing would offer a comparably cost-efficient solution for large-scale metagenomic researches. However, while we have documented the use of the NOPB-based reagent for preservation of samples for metagenomic whole-genome sequencing, it remains to be established whether the NOPB-based reagent is compatible with storage of samples for metabolomics, metatranscriptomics, and metaproteomics.

Moreover, since split fecal samples were used in this study, the volume of samples was small enough to sufficiently mix with the NOPB-based reagent; we did not derive any conclusions about storing large amount of fecal sample by the methods mentioned above. Therefore, we recommend using more than twice volume of NOPB-based reagent for the fecal sample and shake the tube gently to mix the sample and reagent after sampling.

## Conclusions

To solve the problem of preservation and transportation of fecal sample at room temperature, we developed a novel protocol based on NOPB and tested its performance by deep metagenomic sequencing. Our results show that the method can be used for easy collection and storage of fecal samples for 7 days or even longer at room temperature, even when the samples need to be transported using normal commercial routes.

This method will improve the reliability and reproducibility of metagenomic studies using fecal samples collected in remote areas and developing countries and reduce the costs of such studies.

## Additional files

Additional file 1: Supplementary figures. (DOCX 2387 kb)

Additional file 2: Profile tables. (DOCX 14 kb)

## Abbreviations

FDR: False discovery rate
NOPB: *N*-Octylpyridinium bromide
PBS: Phosphate buffer saline
PCoA: Principal co-ordinate analysis
RT: Room temperature
SE: Single-end
