# Supplementary material for: A novel affordable reagent for room temperature storage and transport of fecal samples for metagenomic analyses
Source: Microbiome. 2018 Feb 27;6:43. doi: 10.1186/s40168-018-0429-0 (PMC5828344; doi:10.1186/s40168-018-0429-0)
Supplement: Supplementary file 1 — Supplementary figures. (DOCX 2387 kb) [file 40168_2018_429_MOESM1_ESM.docx]

**
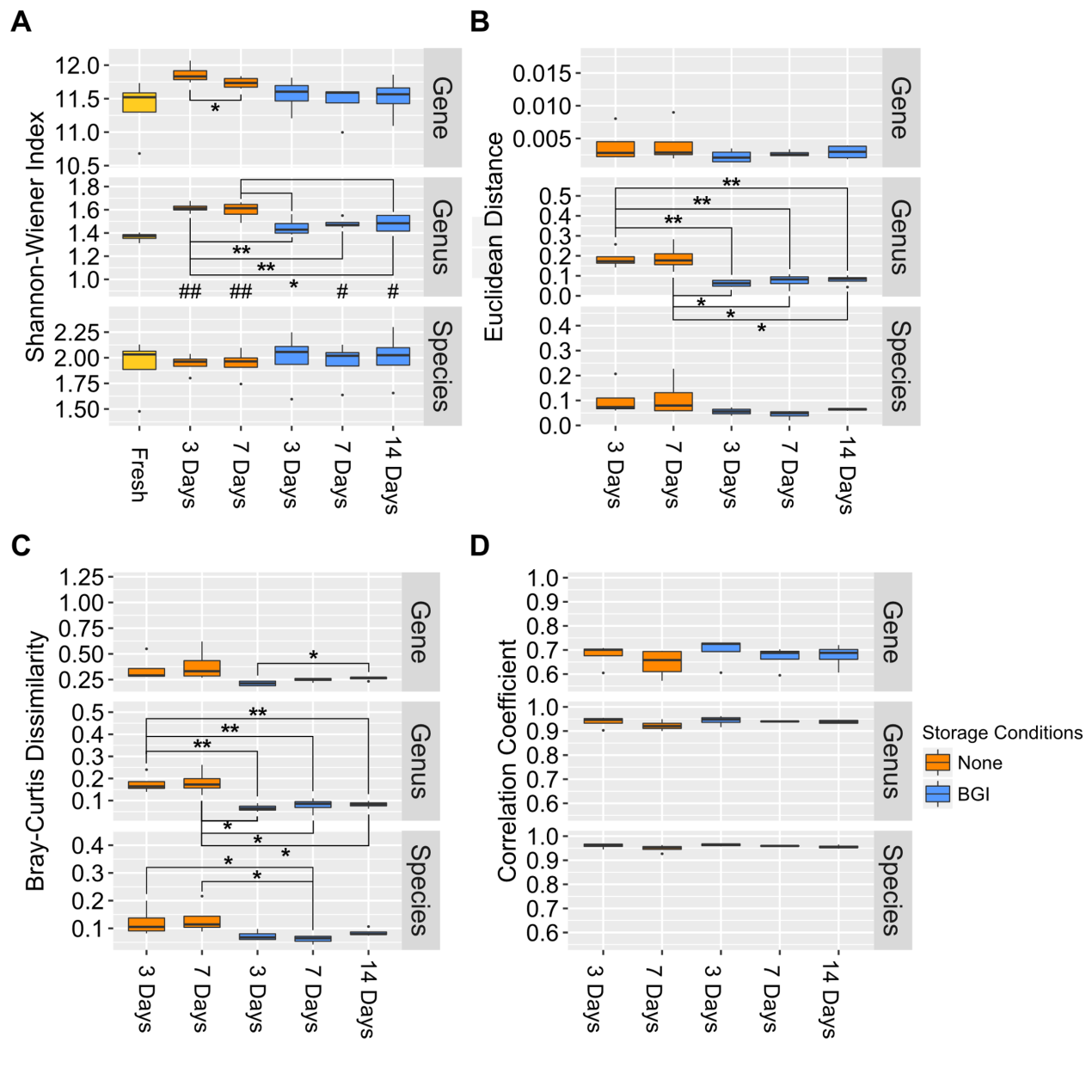
**

**Figure S1 Correlation, dissimilarity and change in α-diversity between NOPB stored and fresh fecal samples on BGISEQ-500.** Sequencing data (Dataset B) used to plot this figure was generated by using the BGISEQ-500 platform. (**A**) shows the α-diversity of the freshly extracted samples and corresponding stored samples. (**B**), (**C**) and (**D**) show the Euclidean distance, Bray-Curtis dissimilarity and Spearman correlation coefficient between the stored and corresponding freshly extracted samples. * indicates significant difference (*p* < 0.05, paired t-test) and ** indicates highly significant difference (*p* < 0.01, paired t-test). # and ## in (**D**) indicate significant difference (*p* < 0.05, paired t-test) and highly significant difference (*p* < 0.01, paired t-test), respectively, in α-diversity in comparison to the corresponding fresh aliquots (n = 4).


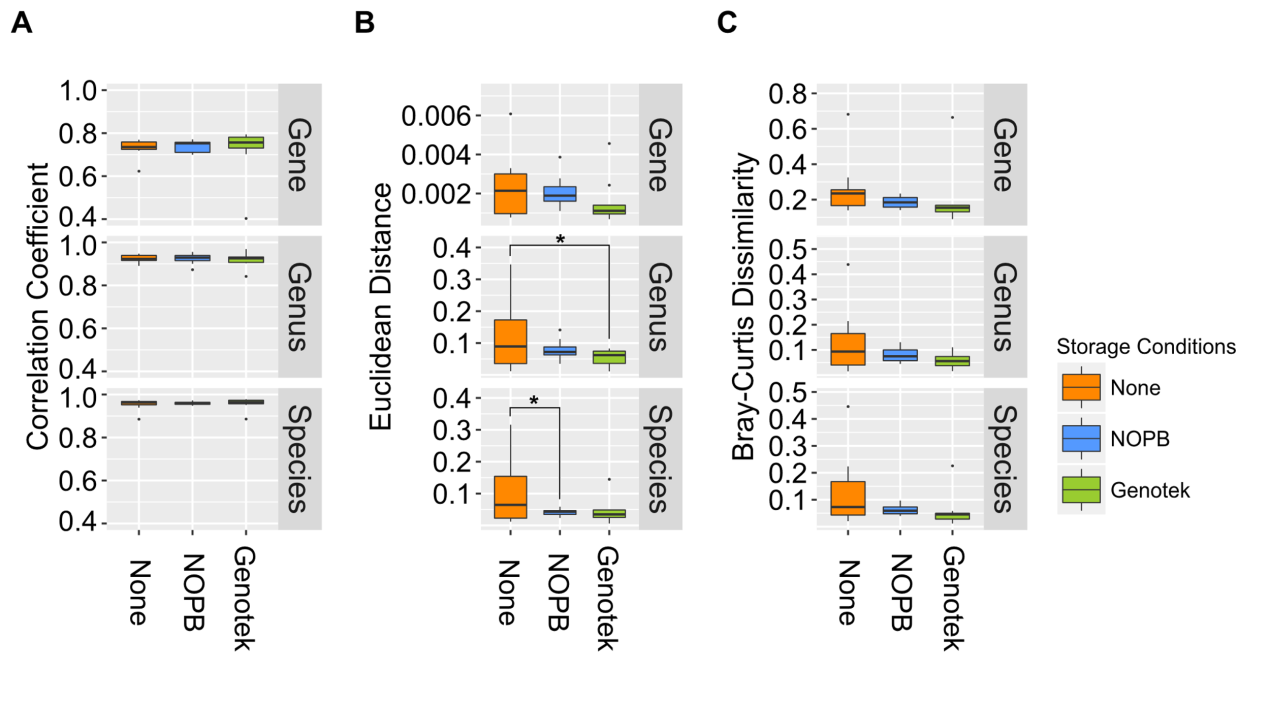


**Figure S2** **Correlation and dissimilarity between fecal samples stored for different duration.** Dataset A was used for comparison between aliquots stored for 3-day and 7-days, respectively. * indicates significant difference (*p* < 0.05, paired t-test). (n = 10)


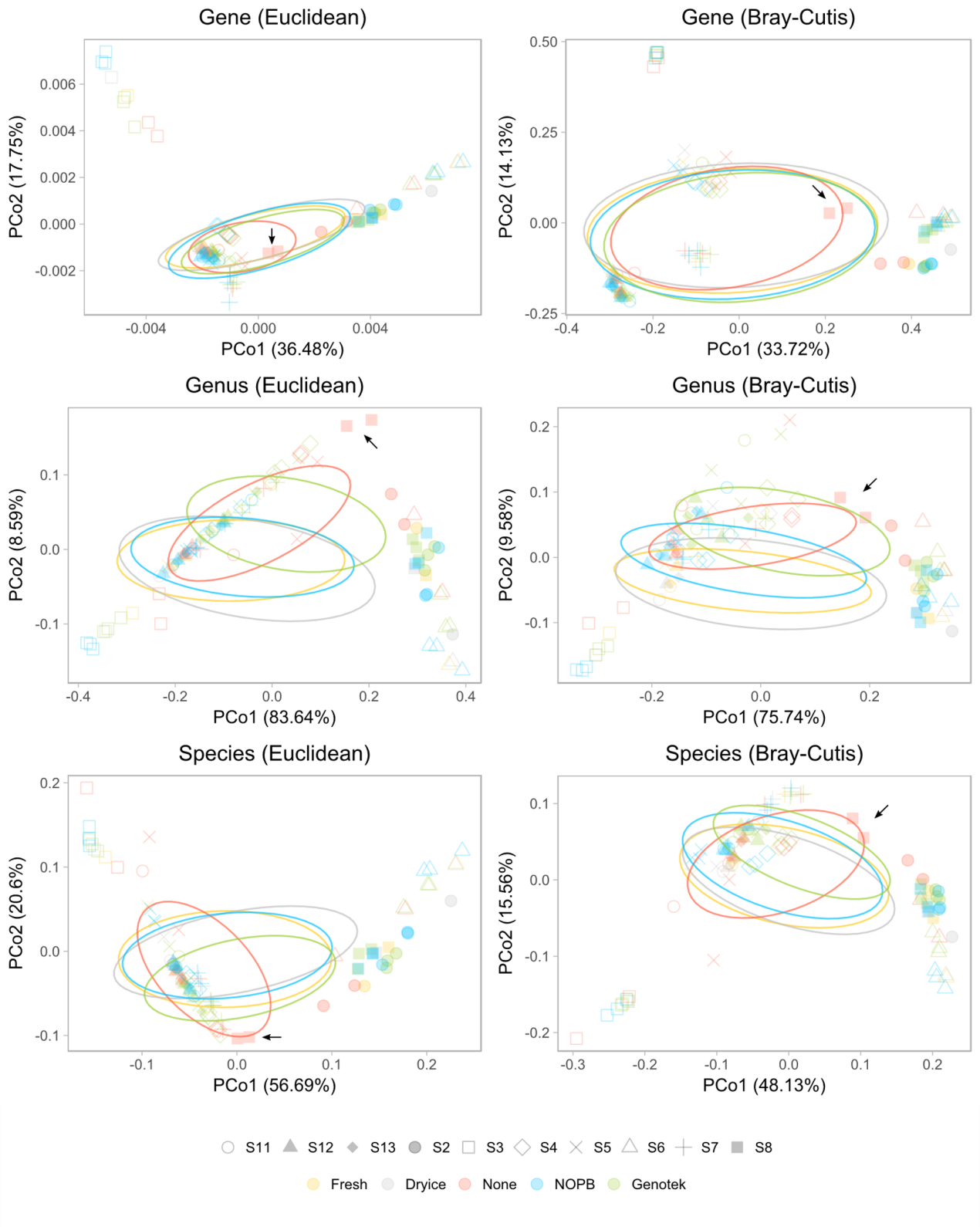


**Figure S3 PCoA comparing different preservation methods.** Different shapes of dots indicate different samples. S11, S12 and S13 originated from the same original sample. Different colors indicate different tested preservation/storage conditions. Aliquots distinguished from the corresponding fresh ones were pointed by arrows.


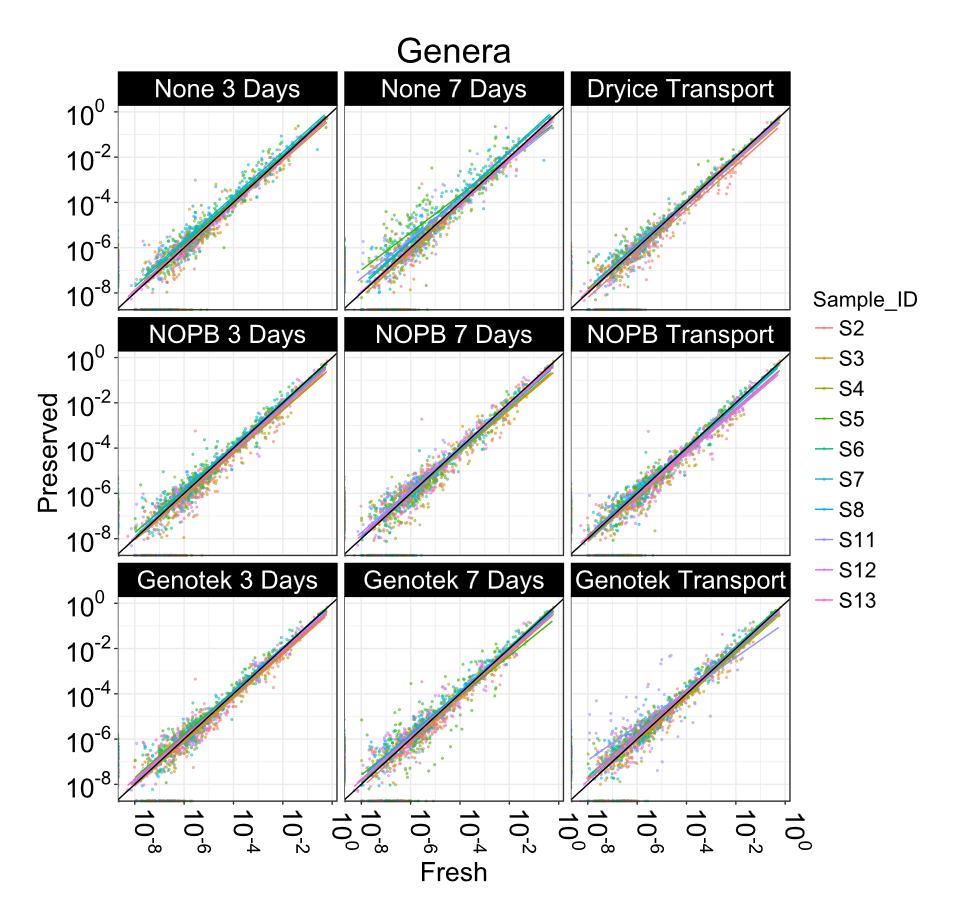


**Figure S4 Relative abundance of genera in stored and corresponding fresh samples.** Dots indicate genera, and straight lines are linear trendlines of each sample.


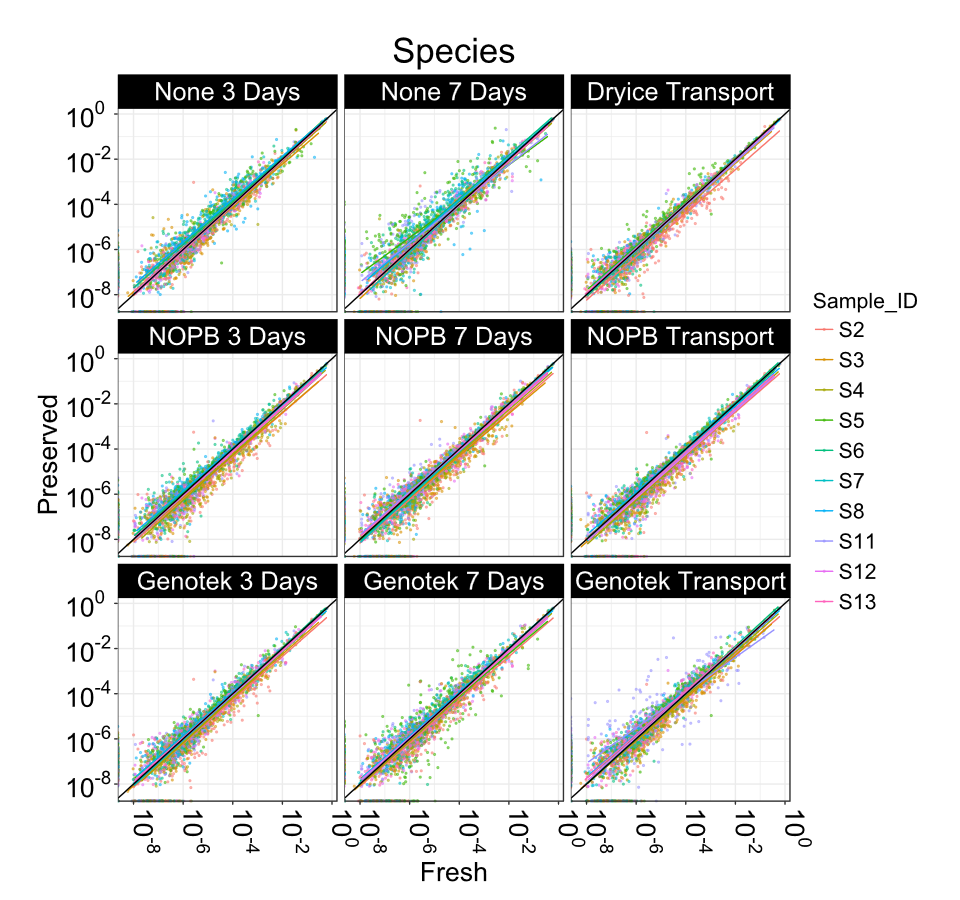


**Figure S5 Relative abundance of species in stored samples and the corresponding fresh samples.** Dots indicate species, and straight lines are linear trendlines of each sample.
