# Supplementary material for: A novel affordable reagent for room temperature storage and transport of fecal samples for metagenomic analyses
Source: Microbiome. 2018 Feb 27;6:43. doi: 10.1186/s40168-018-0429-0 (PMC5828344; doi:10.1186/s40168-018-0429-0)
Supplement: Supplementary file 2 — Profile tables. (DOCX 14 kb) [file 40168_2018_429_MOESM2_ESM.docx]

Dataset A, Profile at genes level:

<https://1drv.ms/u/s!Au6eY4D1BJHdwfBOJa00dm4yQoqZJA>

Dataset A, Profile at species level:

<https://1drv.ms/u/s!Au6eY4D1BJHdwfBK952V6puNt-kZ9A>

Dataset A, Profile at genus level:

<https://1drv.ms/u/s!Au6eY4D1BJHdwfBLjoiujYYvAKsbww>

Dataset B, Profile at genes level:

<https://1drv.ms/u/s!Au6eY4D1BJHdwfBNbleJ2eVtUV5i9Q>

Dataset B, Profile at species level:

<https://1drv.ms/u/s!Au6eY4D1BJHdwfBMIpxp1Px7Vj-Y4w>

Dataset B, Profile at genus level:

<https://1drv.ms/u/s!Au6eY4D1BJHdwfBJ69Ruo8LKB5CgSQ>
